# Assessment of Physical-Chemical Characteristics of Water and Sediments from a Brazilian Tropical Estuary: Status and Environmental Implications

**DOI:** 10.1100/2012/676173

**Published:** 2012-03-12

**Authors:** Madson de G. Pereira, Marta V. A. S. de Andrade, Vanessa C. Ornelas, Raimunda A. N. de Almeida, Maurício P. F. Fontes, Joselito N. Ribeiro, Araceli V. F. N. Ribeiro, Arnaud V. dos Santos, Adriana N. Souza, Claudiane B. de Araújo, Ana C. B. de Araújo, Cássia R. E. Onofre, Maria das G. A. Korn

**Affiliations:** ^1^Departamento de Ciências Exatas e da Terra, Universidade do Estado da Bahia, Rua Silveira Martins 2555, Cabula, 41150-000 Salvador, BA, Brazil; ^2^Departamento de Solos, Universidade Federal de Viçosa, Avenida PH Rolfs, Campus Universitário, 36570-000 Viçosa-MG, Brazil; ^3^Centro de Ciências da Saúde, Universidade Federal do Espírito Santo, Avenida Maruípe, S/N Maruípe, 29042-751 Vitória, ES, Brazil; ^4^Diretoria de Graduação, Instituto Federal de Educação, Ciência e Tecnologia do Espírito Santo, Avenida Vitória 1729, Jucutuquara, 29040-780 Vitória, ES, Brazil; ^5^Instituto de Química, Universidade Federal da Bahia, Rua Barão de Geremoabo 147, 40170-115 Salvador, BA, Brazil

## Abstract

The environmental quality of the Jacuípe River's estuary (very important in northeastern Brazil) was assessed during 2007 and 2008. In water, concentrations (mg L^−1^) of NO_2_^−^ (<0.004 to 0.016), NO_3_^−^ (0.01 to 0.33), soluble PO_4_^3−^ (<0.02 to 0.22), dissolved oxygen (3.9 to 9.6), total contents (mg L^−1^) of Cd (<0.001), Cu (<0.01), Pb (<0.01), and Zn (<0.1), pH (5.60 to 8.00), and electrical conductivity (0.12 to 48.60 mS cm^−1^) agreed with environmental standards. In sediments, clay and total organic matter (%, m/m) varied, respectively, from 8.8 to 12.0 and from 1.1 to 8.8, while infrared, thermogravimetric profile, electronic micrograph, as well as X-Ray analyses showed desirable adsorptive characteristics. However, maximum exchangeable levels (mg kg^−1^) of Cd (1.3), Cu (44.6), Pb (35.7), and Zn (43.7) and their respective maximum pseudototal concentrations (mg kg^−1^): 19.4, 95.1, 68.2, and 30.3 were below the recommended limits. In this sense, it was possible to demonstrate good environmental preservation even with the growing number of industries and touristic activities in the evaluated estuarine area.

## 1. Introduction

Almost 12% of the total fresh water of the planet is in Brazil, and preservation of this huge hydric variety is one of the greatest environmental challenges for this country. In Brazil, Bahia State stands out not only due to its size (564.692.67 km^2^), but also because of the presence of important and numerous rivers, besides being the state that has the largest seashore in Brazil, with 1,183 kilometres in total.

 In the aquatic ecosystem ensemble present in Bahia, the estuaries deserve special attention because of their importance for the procreation of several marine organisms and their natural susceptibility to environmental disequilibrium [1]. Moreover, the tropical estuarine areas are formed by mangroves, which are one the most complex, productive, and fragile ecosystems of the planet. Along the whole of Bahia's coastline extension, the north region seems especially vulnerable due to the presence of one of the biggest industrial complexes in Latin America, and because of the population from the Salvador metropolitan area, with more than three million people.

 In this sense, Bahia's north coast needs constant monitoring for hydric quality, with especial attention for the Jacuípe River's estuary. This river has an extension of 141 km and its mouth is located in Camaçari, near an industrial area where there are several industries, from car manufacture to pharmaceutical factories. Prior to its mouth, the Jacuípe River runs across agricultural areas. In spite of the ecological importance of the Jacuípe River's estuary and its intense use, the last large-scale investigation regarding environmental conditions in this area was carried out in 1989 [2], an 18-year gap regarding the present work. It must be emphasized that this period without research related to hydric quality of the Jacuípe River's estuary corresponded to an accelerated growth in tourism in the area.

 Thus, this manuscript aims to fill the lack of information about the preservation of an area of huge importance for Bahia, ecologically, economically, and socioenvironmentally. In this sense, analyses of water and sediments were carried out, in that the water analysis referred to the total amounts of Cd, Cu, Pb, Zn (in some samples), as well as levels of NO_3_^−^, NO_2_^−^, and soluble PO_4_^3−^. Additionally, pH, temperature, electrical conductivity, and dissolved oxygen measurements were carried out. Sediments were analysed regarding the exchangeable and pseudototal (in some samples) amounts of Cd, Cu, Pb, and Zn, total percentages of organic matter, granulometric composition, and morphology, as well as infrared, thermogravimetric, and X-ray diffraction analyses. Metal selection was based on use and/or toxicity criteria, especially for cadmium and lead, which do not show any essentiality for animals or vegetables [1, 3–6].

## 2. Experimental

### 2.1. Description of the Area

**Figure 1:**
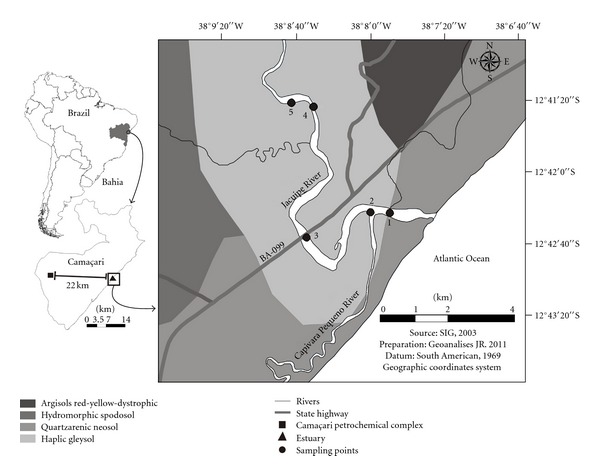
Description of the investigated area with sampling points.

Figure 1 contains a general description of the areas researched, as well as the localisation of the five sampling points for water and sediments. These points are related to locations along the Jacuípe River from its mouth (approximate distance of 5 km from the first to fifth point). During the sampling periods (in 2007 and 2008), the two first points (strong oceanic influence and around areas of aquatic sports) had little vegetation. Point 3 was located beneath a bridge with heavy vehicle traffic and around residential and commercial settlements, while points 4 and 5 were located in areas with dense mangrove vegetation. The entire estuary zone is within the boundaries of the Capivara environmental protected area, limited by the Jacuípe River in the north and by the Atlantic Ocean to the east; there is a state-owned highway to the west, with heavy traffic, and an inorganic chemical products factory in the south. Additionally, the Camaçari industrial complex is located 22 km from the described region.

The researched estuary area has a humid tropical weather with average annual temperatures near 25°C and rainfall levels of up to 1500 mm. In hydrological terms, the Jacuípe River average flux is of 21.8 m^3^ s^−1^ at its mouth, controlled by the Santa Maria dam 40 km away, and influenced by the Capivara Pequeno and Capivara Grande affluents, downstream of the dam [7].

The most common soils found at the Jacuípe River's mouth are characterised by the translocation of sesquioxides of aluminum and iron, phyllosilicate clays, besides organic matter. The profusion of this kind of soil in Camaçari agrees with the existence of large quantities of sandstones, a type of sedimentary rock [8].

## 3. Reactants and Equipments

All the reactants used in the experiments were of analytical degree, Merck brand (Germany), or Vetec (Brazil). The preparation of solutions was carried out with ultrapure water (0.05 *μ*S cm^−1^) from a Gehaka brand (Brazil) purifier. Stock solutions of metals (Cd, Cu, Pb, and Zn) at 1,000 mg L^−1^ were obtained from Qhemis (Brazil) with certificates of traceability.

 Temperature, pH, electrical conductivity, and dissolved oxygen measurements were conducted with portable probes from the following manufacturers and models, respectively: Phtek (Brazil) pH-100, Phtek (Brazil) CD-203, and Lutron (USA) DO-5510. A table of horizontal agitation Nova Ética (Brazil), model 109, was used to extract the exchangeable levels of Cd, Cu, Pb, and Zn from the sediments. Pseudototal extractions of sediments and total decompositions of water samples were carried out with a digestion block Marconi (Brazil), model MA-4025.

 Determination of total amounts of the metallic species in water and exchangeable amounts in sediments were completed by inductively coupled plasma optical emission spectrometry (ICP OES), with Varian brand (Australia) equipment, model Vista-RL. The wavelengths employed for the Cd, Cu, Pb, and Zn were 214.439, 327.395, 220.353, and 213.857 nm, respectively. The optical emission spectrometer was operated with measurement power of 1300 W and 40 MHz of radiofrequency. Calibrations were carried out on a daily basis, from successive dilutions of a standard mixed solution at 1.000 mg L^−1^.

 The flame atomic absorption spectrometry (FAAS) was employed for quantifying the pseudototal amounts of Cd, Cu, Pb, and Zn in the sediments, using a Varian (Australia) spectrometer, model SpectrAA 220. In all the analyses, an air-acetylene flame was used; wavelengths for Cd, Cu, Pb, and Zn were 214.438, 324.754, 220.353, and 213.856 nm, respectively. Standard solutions at 1.000 mg L^−1^ were used for the daily preparation of calibration curves.

 X-ray analysis of sediment samples were performed by means of a Rigaku (Japan) diffractometer, model X'Pert Pro PW 3040/60, while a Jeol electron microscope (Japan), model JSM-6610LV, was employed for obtaining micrographs of the sediment particles. The thermogravimetric profile of the sediment was obtained with a TA Instruments Universal V2.3C thermogravimetric analyzer (USA), while a Perkin-Elmer Spectrum 100 infrared spectrometer (USA) was used for identifying functional groups in the sediment.

### 3.1. Sampling Procedures

Water and sediment samples were collected at the five points shown in Figure 1 and, for this purpose, the Civil Defense of Camaçari provided a boat. The sampling periods were defined according to the tide table, always prioritising the ebb tide periods. For water, 13 sampling campaigns were carried out, while 8 campaigns were conducted for sediments (Table 1). 

 Water samples were collected from the surface, and at a depth of 4 m by immersing polyethylene bottles. A Bailer type collector was used specifically for the deep samples. All the collectors were previously decontaminated using a solution of HCl 10% (v/v) and washed in ultrapure water.

 After sampling of almost 1 L of water from the surface and from depth, the volume was divided into two polyethylene bottles, which are stored in foam boxes with ice. In the laboratory, one of the bottles was refrigerated up to 4°C, until determination of the soluble phosphate, within a time limit of 72 h. The second bottle was immediately used for determining nitrite and then refrigerated at 4°C for quantifying nitrate within a time limit of up to 48 h [9].

### 3.2. Procedures of Analyses

All procedures described below were rigorously performed according to standard protocols, which are applicable to environmental analyses related to this work. Furthermore, the determination of metals in all matrices (sediments—pseudototal and exchangeable fractions and water), as well as the quantification of anions in water samples, was carried out in routine/research analytical laboratories subject to constant verification of the quality of results, including the use of certified materials.

#### 3.2.1. Water

During the thirteen sampling campaigns, 65 surface and 65 deep water samples were analysed. Spectrophotometric analyses of the 130 samples were carried out in triplicates, with analytical blanks. Measurements of pH, electrical conductivity, dissolved oxygen, and temperature were done once only, and the two latter quantifications were only performed on surface water samples.

Nitrite determination was carried out in accordance with the Griess method, by means of the reaction of this anion with sulphanilamide and chlorhydrate of N-(1-naphthyl)-ethylenediamine (NED), with a further quantification at a maximum wavelength of 543 nm and in a buffered media at pH 8.5. For nitrate quantification, a procedure of nitrite reduction was firstly adopted; by percolating the samples and standards through a 25.00 mL burette containing copperized Cd [9]. After nitrate reduction, the nitrite determination was realised as previously described. Determination of the soluble phosphate levels employed the molybdenum blue spectrophotometric method at a maximum wavelength of 680 nm, based on the ammonium molybdate and orthophosphate reaction, in acid medium, catalysed by antimony potassium tartrate [9]. When necessary, water samples were submitted to quantitative filtration. Standards for NO_2_^−^, NO_3_^−^, and PO_4_^3−^ were, respectively, obtained in the following concentration ranges: 0.05 to 1.0, 1.0 to 10.0, and 1.0 to 5.0 mg L^−1^. These standards were prepared from NaNO_2_, NaNO_3_, and KH_2_PO_4_ and the determinations were realised in triplicates and with analytical blanks.

 The pH, temperature, electrical conductivity, and dissolved oxygen determinations were made by direct immersion of portable probes after the required calibration procedures.

 Total amounts of Cd, Cu, Pb, and Zn were quantified in surface water samples collected during July, August, September, and October of 2007 and March of 2008. For this purpose, the samples (in triplicate and with analytical blanks) were submitted to a previous preconcentration of 5X by means of an evaporation step at 60°C. After that, the samples were digested with HNO_3_, according to a standard procedure [9]. In this procedure, samples with heavy loads of suspended solids were filtered with quantitative paper and the filtered fraction (100 mL) was transferred to erlenmeyers, covered with watch glasses, which are heated at 100°C on a hot plate. In a next step, 5 mL of HNO_3_ 14 mol L^−1^ were added and the heating was maintained until attaining a translucid liquid phase, corresponding to volumes of about 10 mL. After this, the volume was measured to 100 mL with ultrapure water and stored in polyethylene bottles, refrigerated at 4°C until FAAS analyses.

 The sample residual acidity was determined and used for normalising the metal standard solutions acidity in order to avoid differences in viscosity. The standards were prepared from individual stock solutions (at 1000 mg L^−1^) in the following concentration ranges (in mg L^−1^): 0.1 to 1.0 (Cd), 0.5 to 10.0 (Pb), and 0.5 to 5.0 (Cu and Zn). The acidity correction and concentration ranges of the standards were also maintained for quantifying Cd, Cu, Pb, and Zn in the sediments as described below.

#### 3.2.2. Sediments

Sediments were sampled at the riverside, in each one of the five sampling sites specified in Figure 1, totalling 40 samples during the eight months of monitoring. Sampling was manually conducted; sediments were stored in plastic bags and placed in a foam box with ice. In the laboratory, samples were dried in a stove at 80°C, until reaching a constant mass and sieved in a 0.053 mm mesh sieve in order to determine the exchangeable and pseudototal concentrations of Cd, Cu, Pb, and Zn.

 Extractions of the exchangeable amounts of Cd, Cu, Pb, and Zn in the sediments collected during the eight sampling campaigns were completed in 5 replicates, also with analytical blanks. For this, almost 1 g of previously dried sediment (0.053 mm) was agitated with 20.00 mL of HCl solution at 0.1 mol L^−1^ (200 rpm), for 2 hours [10], and at room temperature of around 28°C. After agitation, supernatants were filtered and transferred to previously decontaminated polyethylene bottles, which are refrigerated (4°C) for posterior ICP OES analyses.

 Sediments collected during the sampling campaigns of 09/2007, 03/2008, 06/2008, and 07/2008 were also submitted to quantification of the pseudototal amounts of Cd, Cu, Pb, and Zn, also in 5 replicates and with analytical blanks. The method described by the Environmental Protection Agency and previously cited [10] was used. Thus, 500 mg of the samples were placed in digestor tubes, then 15 mL of HNO_3_ 14 mol L^−1^ were added. After a 12 h inactive period, tubes were heated at 160°C for 4 h, with a further addition of 8 mL of H_2_O_2_ 30% (v/v), maintaining the temperature at 160°C for 30 more minutes. Then, the digested products were filtered for separating silica; they were also adjusted with ultrapure water to obtain 50.00 mL and refrigerated at 4°C until FAAS analyses.

Sediments sampled in 03/2007 were heated (triplicates) in a furnace at 550°C for 4 h, and the mass difference was used to calculate the total concentration of organic matter [2]. Clay percentages of these samples were obtained by the combined sieving procedure recommended by the Brazilian Technical Standards Association (ABNT, in Portuguese) [11].

 For X-ray diffraction, the sediment sample collected in 03/2007 (point 3) was used. This sample was firstly submitted to granulometric fractioning [11] in order to obtain the silt fraction. In a next step, this fraction was exposed to X-rays (radiation Co-K*α*, *λ* = 1.79026 Å) with the 2*θ* angles varying within 5 to 50°. The applied voltage and current were 40 kV and 30 mA, respectively. In order to identify functional groups, the sediment obtained from sampling point 5 (03/2007) was characterised by Fourier transform IR spectroscopy using KBr discs and varying the spectral range from 4,000 to 400 cm^−1^. Infrared analysis was also made with impregnated sediments, where 2.0 g of the sample collected at point 5 (03/2007) were mechanically agitated (200 rpm) with 50.00 mL of cadmium and lead solutions at 10 mg L^−1^ and at pH 6.0, for 2 h. In a next step, supernatants were discharged and the sediment was heated at 60°C for 24 h with posterior infrared analysis [12].

 For electron microscopy, sediment collected at point 5 (03/2007) was covered with a thin layer of gold and an electron acceleration voltage of 20 kV was applied. Thermogravimetry of this same sample was performed by heating sediment particles from 25°C to 1,000°C at 10°C min^−1^, in an oxidant atmosphere.

## 4. Results and Discussion

### 4.1. Water Analyses

Table 2 contains the results of physical-chemical parameters of water samples.

**Figure 2:**
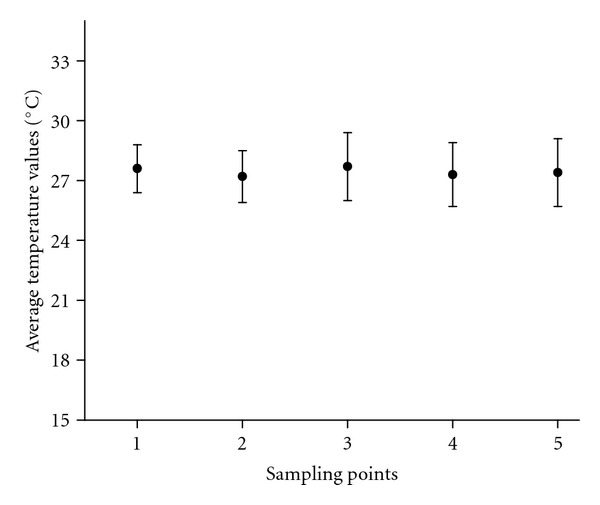
Average water temperature for all of the sampling points.

**Figure 3:**
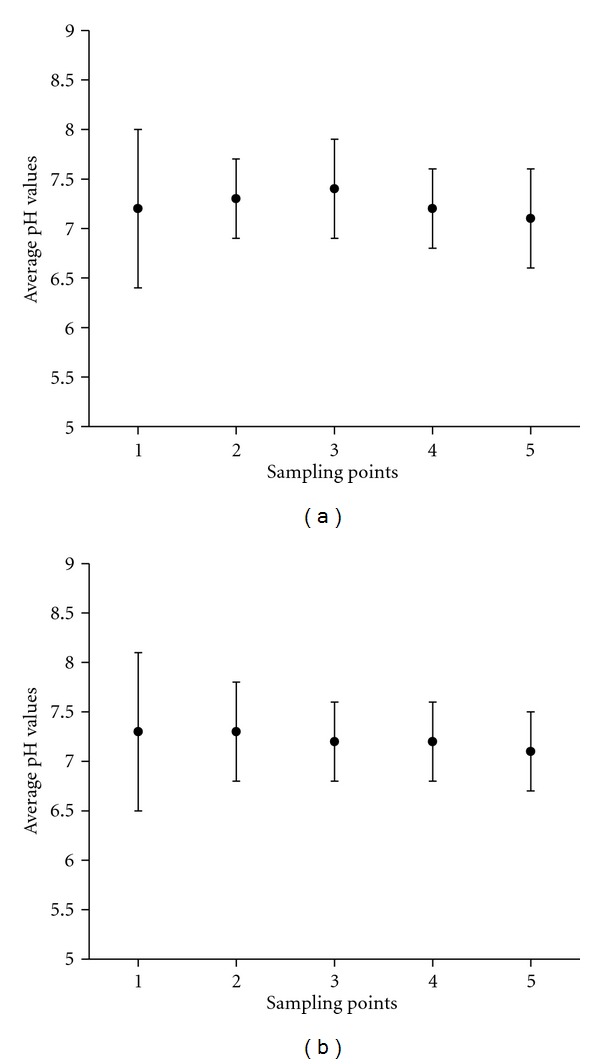
(a) Average pH (surface water) for all of the sampling points. (b) Average pH (deep water) for all of the sampling points.

The average temperature distribution during the 13 sampling campaigns (Figure 2) was consistent with the expected seasonal variations for humid tropical regions. The deviations were calculated by considering all of the thirteen results obtained for each sampling point. The same reasoning was also applied to the deviations shown in Figures 3(a), 3(b), and 5.

As shown in Table 2 and Figures 3(a) and 3(b) (average values), the vast majority of the 130 pH results from surface and deep samples was found in the range established by the National Environment Council (6.0 to 9.0), resolution number 357 [13]. The normality in the pH shows the absence of significant sources of acidic or alkaline compounds in the evaluated area, or immediately upstream thereof. In addition, the volume of water flowing through the five sampling points seems high enough to dilute any punctual pollution source able to modify the natural conditions of pH.

Electrical conductivity levels found in different river mouths can only be compared if these areas belong to regions with similar climatic and geographical characteristics. In this context, the conductivities reported in this study were compared with those of the Formoso River mouth [14] in Pernambuco, another state of northeastern Brazil. The mouth of this river had conductivities of up to 45.0 mS cm^−1^ at the points closest to the ocean, which is similar to points 1 and 2 (Figure 1).

**Figure 4:**
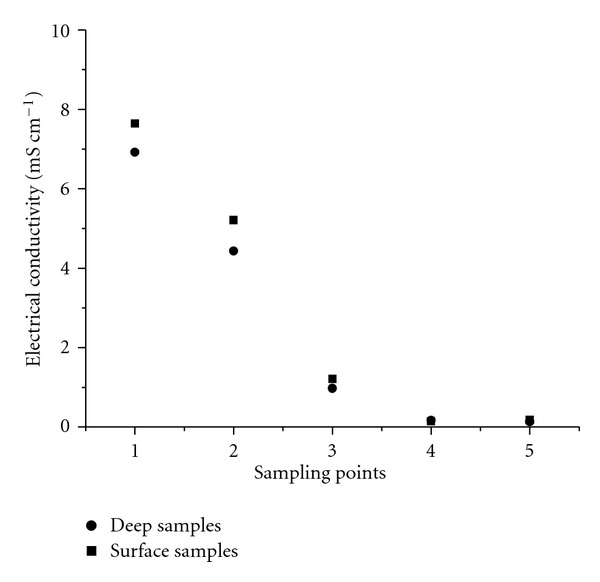
Electrical conductivity in water samples collected in July of 2007.

 At the mouth of the Jacuípe River, a tendency towards a decrease in electrical conductivity after the first sampling site was observed in most cases. Moreover, significant differences between the conductivity of surface and deep samples were not identified and, when present, such differences occurred at points 1 and/or 2, as can be exemplified by the samples collected in 08/2008. This behaviour is explained by the higher density of seawater and its incomplete mixture with freshwater.  Figure 4 illustrates the trend for a single collection (07/2007) that reflects the general behaviour observed throughout this work, that is, a steep drop in electrical conductivity after the first sampling site and the similarity between surface and deep water.

Still in comparative terms, Silva et al. (2010) [15] recorded a large amount of data on electrical conductivity at the São Francisco River's mouth, one of the most important in northeastern Brazil. When compared to the mouth of the Jacuípe River, the electrical conductivity values were significantly higher in the mouth of the São Francisco River, as a result of numerous saline flows along much of its watershed, such as those originating from farming activities.

**Figure 5:**
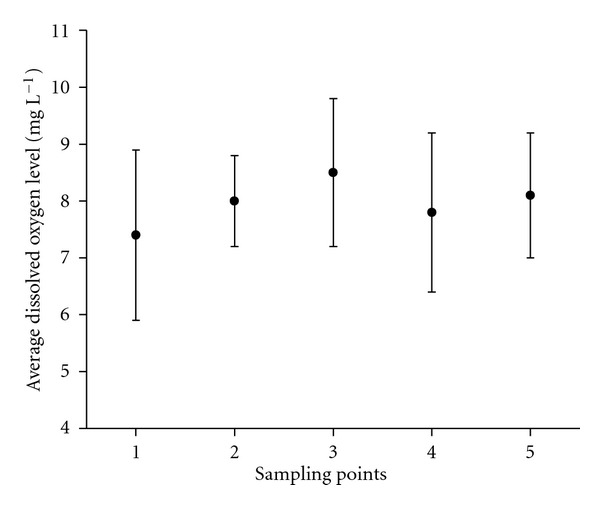
Average dissolved oxygen concentrations (surface water) for all of the sampling points.

All the levels of dissolved oxygen throughout the 5 sampling points were above the minimum required by Brazilian environmental legislation, which is 5.0 mg L^−1^ for Class 2 water [13]. This result is related to reduced levels of biodegradable material [5] and/or high local rate of reoxygenation. This last statement is based on the high incidence of sunlight and low turbidity (visual observations), which appreciably favours photosynthesis. The average levels of dissolved oxygen during the sampling campaigns and at various collection points are illustrated in Figure 5.

The N-NO_2_^−^ and N-NO_3_^−^ (Table 3) concentrations also agreed, for most samples, with the National Environment Council [13], resolution number 357, which establishes the maximum limits of 1.0 and 10.0 mg L^−1^, respectively, for nitrite and nitrate in Class 2 water. These results are coherent with the considerations of low levels of biodegradable organic matter, given that these two anions may be derived from the aerobic decomposition of nitrogenated molecules. The weathering of rocks, soil leaching, and/or erosion are also responsible for the addition of NO_3_^−^ to the river and consequently estuary environments [8], the two latter sources being of great importance to rivers in areas with intense agricultural activities. From the results obtained, it can be concluded that neither of the possible introductory sources mentioned above was important enough to promote the accumulation of nitrate in the investigated estuarine area.

The phosphate comprises another anion of great importance for assessing the quality of an aquatic ecosystem, as it can be responsible for eutrophication, along with nitrate [1]. According to Table 3, only two samples had soluble phosphate levels above the maximum allowed by Brazilian environmental legislation [13], which is 0.15 mg L^−1^ for Class 2 water. In addition to the natural weathering process of rocks and anthropic inorganic fertilizers, phosphate is predominantly introduced into bodies of water through domestic and industrial discharges containing synthetic detergents [1]. Thus, the normality in P-PO_4_^3−^ concentrations (Table 3) reinforces the absence of significant volumes of domestic discharges in the studied area, as well as in the nearby areas upstream. It is worth mentioning that, although there is a large industrial complex approximately 20 km from the assessed area (Figure 1), there is no evidence of phosphate pollution from this industrial conglomerate.

**Figure 6:**
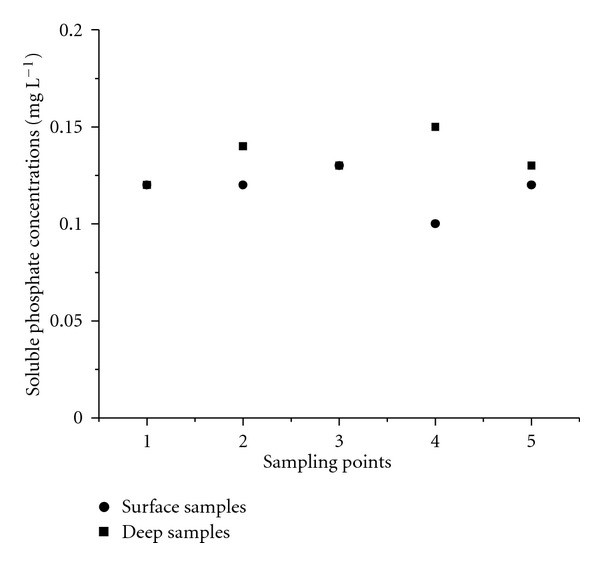
Soluble phosphate concentrations in water samples collected in July of 2007.

For the three quantified anions (NO_2_^−^, NO_3_^−^, and PO_4_^3−^), the averages of surface and deep samples were similar over the entire sampling period, and the dispersions between them were also relatively close. This trend towards uniformity in concentrations of dissolved ionic species can be explained by the absence of localised points of evictions beyond homogenization promoted by the river's flow and current.  Figure 6 illustrates the general behaviour observed for the anions between surface and deep samples. For the sake of simplicity, only the concentrations of soluble phosphate in one sampling campaign (07/2007) were considered.

All samples of surface water collected from 5 sampling campaigns (July to October of 2007 and March of 2008) revealed levels of cadmium, copper, lead, and zinc below the method's detection limit: 0.001 (Cd); 0.01(Cu); 0.01(Pb); 0.1(Zn) mg L^−1^, which are lower than the maximum allowed by Brazilian environmental legislation [13]. This result suggests the absence of discharges containing high concentrations of Cd(II), Cu(II), Pb(II), and Zn(II) during the cited samplings. However, it should be emphasised that the analysis of water only provides a momentary panorama of the river's environmental condition, and these results should be correlated with other parameters, such as morphological and chemical characteristics of the sediments.

### 4.2. Sediment Analyses

Table 4 contains the exchangeable concentrations of cadmium, lead, copper, and zinc in the sediment samples.

For cadmium, exchangeable levels in some samples were found to be very close to the maximum recommended [16], which is 1.0 mg kg^−1^. Although this metal has been identified in a small number of samples, indications of buildup causes concern due to its high toxicity and because these sediment samples are surface, suggesting recent sedimentation. The retention of alarming exchangeable levels of cadmium may be related to the leaching of soil particles containing certain types of fungicides employed in agriculture. This argument is valid because the sediments presenting cadmium buildup were collected during the rainy season, a period in which erosion and leaching are often observed.

Although the presence of lead is equally troubling, all of the exchangeable concentrations encountered were below the maximum recommended [16], which is 132.0 mg kg^−1^. Lead was detected at diverse periods of the year relating to rain occurrence, and this variability hiders the association with transport mechanisms through soil leaching and/or erosion, as discussed for cadmium. According to Bowen (1979) [17] and Cox (1997) [18], the relative position of lead (36th) in the Earth crust far exceeds that of cadmium (65th), and this may explain naturally larger buildups of lead in geological matrices such as rock fragments, minerals, and soils that ultimately compose the sediments.

Despite the industrial development present in the Jacuípe River's estuary and widespread use of copper and zinc, the exchangeable concentration of both metals in sediments did not exceed the maximum recommended limits of 73 (Cu) and 145 (Zn) mg kg^−1^ [16]. Thus, the levels found in sediments can be attributed in a large part to the ample distribution of both metals in the earth's crust: 24th and 25th position for Cu and Zn, respectively [19]. Although the Capivara Pequeno River has been influenced by the operation of a copper ore processing plant for several years, the sediments collected at point 2 (where this river flows into the Jacuípe River—Figure 1) did not present alarming exchangeable levels of this element.

The pseudototal levels of Cd, Cu, Pb, and Zn in some of the estuarine sediments are listed in Table 5. Although the pseudototal fractions have only been quantified in sediments collected in some few sampling campaigns, it was possible to observe an evident difference between the pseudototal and exchangeable levels of cadmium, copper, and lead. This behaviour can be attributed to the affinity of Cd(II), Cu(II), and Pb(II) with mineral (notably clays) and/or organic components of the sediment particles. On the other hand, this difference was not so evident for zinc, indicating its lower affinity with the components already mentioned. In general, higher pseudototal levels of copper were observed at sampling point 2, exactly at the confluence of the Jacuípe and Capivara Pequeno Rivers. As discussed earlier, this last river received wastes from a copper processing plant.

The differences observed in metal retentions on sediment particles can be partially explained by charge's density of the analytes. Lead ions have the smallest hydrated radius (or highest charge's density) among the other metallic ions, thus showing great adsorptive fostering related to electrostatic retentions. Contrarily, zinc ions present the highest hydrated radius and smallest charge's density, thus justifying its low affinity by the adsorptive sites. For cadmium and copper, intermediate characteristics are observed. Despite considerations about occurrence of electrostatic forces, retentions of the four analytes by specific interactions (e.g., chemical bounds) cannot be disregarded.

From an environmental point of view, a reasonable difference between exchangeable and pseudototal levels is important, because it indicates that an expressive portion of metals will not be assimilate by the local biota, considering only the natural conditions of water bodies. This statement is true, because the metals belonging to the pseudototal fraction are more effectively (or less reversibly) retained since they form, for example, very stable complexes with humified organic matter. Nevertheless, benthic organisms ingest sediment particles and they can be directly contaminated with spillover effects for the entire food chain.

Sarkar et al. (2004) [20] assessed the pseudototal concentrations of Cd, Cu, Pb, and Zn, among other metals, also in surface layers of estuarine sediments collected in northeastern India. In that study, the authors used extraction with aqua regia, and the following results were obtained: Cd (0.1 to 0.2 mg kg^−1^), Cu (21.5 to 64.1 mg kg^−1^), Pb (13.7 to 24.9 mg kg^−1^), and Zn (26.0 to 162 mg kg^−1^). These data were considerably lower than those obtained in this work for cadmium, moderately lower compared to copper and lead, and higher than those recorded for zinc. Given that the investigations [20] were conducted in an area highly impacted by diverse industrial activities, the pseudototal concentrations found in the estuarine sediments of the Jacuípe River are troubling. Despite these relatively high pseudototal concentrations found in the Jacuípe River's sediments, it should be noted that all the results in Table 5 are smaller than the maximum permissible levels for Cd, Pb, and Zn (mg kg^−1^): 30, 4800, and 620, respectively [16]. For copper, 30% of the results exceeded the maximum level set for this element, which is 73 mg kg^−1^ [16].

### 4.3. Structural Characterisation of the Sediments

**Figure 7:**
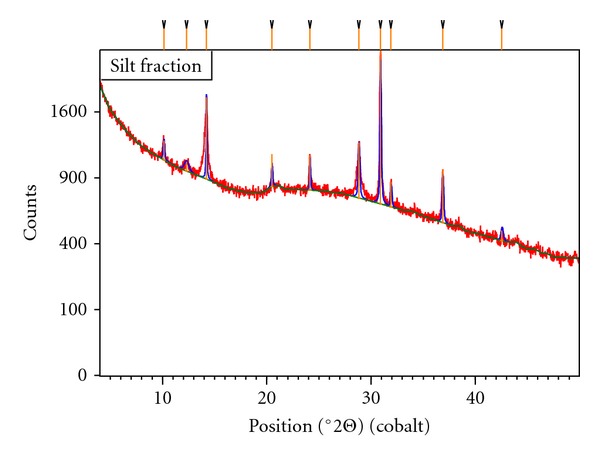
X-ray diffractogram of the sediment collected at sampling point 3 (March of 2007).

With the aim of evaluating the minerals present in estuarine sediments and relating them to the adsorptive capacity, the sediment sample collected at point 3 (Figure 1) in 03/2007 was analysed by X-ray diffraction. The choice of the point 3 was based on its greater capacity of sedimentation, since the third site is located in an area with almost no slope and slow water flow. In this sense, a good representativeness of the local mineralogical composition is expected at this point. The diffractogram obtained (Figure 7) reveals four predominant structures; two primary (quartz and feldspar) and two secondary (illite and kaolinite) minerals. The last two have a great surface area and are responsible for appreciable cationic exchange capacity. The attributions of the 2*θ* angles were conducted according to information contained in the equipment database and are listed in Table 6. Due to the remarkable presence of quartz in the sand fraction of the sediments, it was necessary to remove sandy particles as already mentioned. Otherwise, the signals related to quartz would fade out the peaks of other minerals present in small amounts. So, X-ray diffraction was conducted on the silt fraction, which also represents the minerals contained in the clay fraction. 

The identification of these four major minerals is consistent with the predominant soil types in the area studied, which are described in Figure 1. These soils have a high degree of weathering, very distinct pedogenetic origins, and remarkable differences in organic matter content, as well as quality and quantity of clay. According to Figure 1, from the four major soils of the Jacuípe River's estuary, Haplic Gleysol is predominant at the five sampling points. This soil is originated from sandy deposits, thus justifying the expressive contents of quartz in the sediments. The presence of kaolinite and illite is explained by chemical weathering of primary minerals such as feldspar (KAlSi_3_O_8_), which make up the solid phase of all the soils described in Figure 1. In this sense, hydrolysis of KAlSi_3_O_8_ generates kaolinite (Al_2_Si_2_O_5_(OH)_4_) as can be seen in (1):

(1) $$\begin{array}{cc} & 2\text{KAl}{\text{Si}}_{3}{\text{O}}_{8(\text{s})}+\;2{\text{H}}_{2}{\text{CO}}_{3(\text{aq})}\;+\;{\text{H}}_{2}{\text{O}}_{\left(\text{l}\right)}\\ & \;\to {\text{Al}}_{2}{\text{Si}}_{2}{\text{O}}_{5}{\left(\text{OH}\right)}_{4(\text{s})}+4\text{Si}{\text{O}}_{2(\text{aq})}+{2{\text{K}}^{+}}_{\left(\text{aq}\right)}+2{{{\text{HCO}}_{3}}^{-}}_{\left(\text{aq}\right)}. \end{array}$$

It should be noted that the relation between soil type and the mineral composition of sediments is valid because the erosion of the first is an important source of particles in river beds. Notably, the identification of kaolinite and illite plays an important role in the dynamics of retaining metallic species, due to high surface area and important adsorptive chemical groups, including hydroxyls [21, 22]. The laminar distance of illite is approximately 10 Å, which is larger than hydrated ionic radius of all the metallic analytes, thus offering surface areas able to support effective cationic exchanges [21].

**Figure 8:**
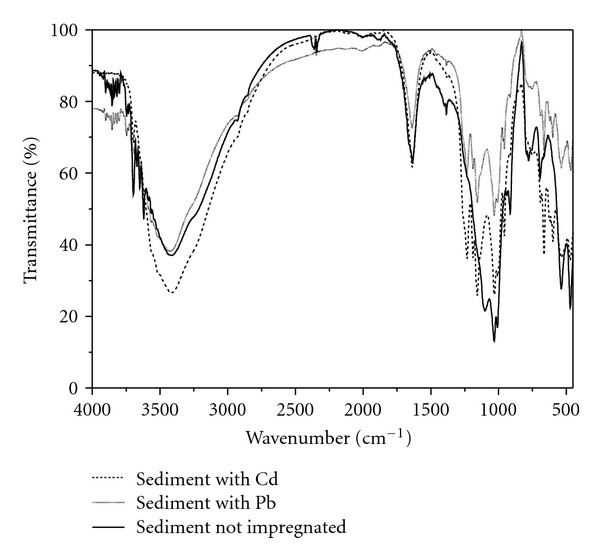
Infrared spectrum of the sediment sample collected at point 5 (March of 2007). Black and continous line is related to sediment not impregnated, while gray and continous line is related to sediment with Pb.

 The clay and organic matter levels of the sediments sampled in 03/2007 are shown in Table 7. The trace amounts of clay at sampling points 1 and 2 are justified by a greater proximity of the ocean and the subsequent transport of sand to these points. Thus, the pseudototal contents of metals in sediments collected from sampling points 1 and 2 are largely associated with organic constituents. As can be seen in the infrared spectrum (Figure 8), the presence and interactions of different chemical organic groups with Cd(II) and Pb(II) corroborate this assumption.

Figure 8 shows broad bands between 3.500 and 3.000 cm^−1^, which can be ascribed to overlap of bands concerned with the stretching vibration of N–H bonds from amines and amides as well as alcoholic and phenolic hydroxyls and carboxylic acids. In the range of 3,000 to 2,850 cm^−1^, the absorption is mainly assigned to the C–H bond from aliphatic groups. Peaks near 1,640 cm^−1^ can be ascribed to the C=O bond stretching of carbonyl groups, while the band in 1,380 cm^−1^ corresponds to stretching of C–O belonging to phenols. The bands between 1,400 and 500 cm^−1^ can be attributed to vibrations of Si–O–Si bonds present in silicates, while vibrations of Si–O–Al are responsible for the bands between 912 and 525 cm^−1^ [23]. When cadmium or lead are present (impregnation experiments), it is possible to verify important spectral modifications both in the intensity and shape of the bands. Ultimately, these changes indicate associations between the metallic ions and organic groups. Impregnations with nickel were not made because the realised tests were sufficient to show similarities between adsorptive sites of the sediments even considering metals with distinct chemical characteristics. These results point out for predominance of nonspecific adsorption phenomena based on electrostatic forces.

**Figure 9:**
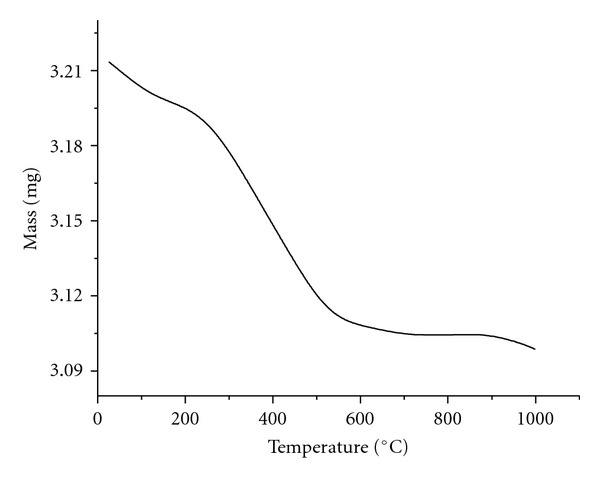
Thermogravimetric profile of the sediment collected at sampling point 5 (March of 2007).

**Figure 10:**
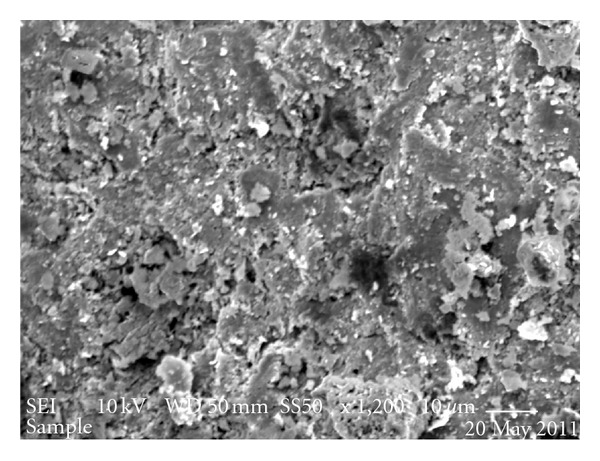
Electron micrograph of the sediment collected at sampling point 5 (March of 2007—Magnification of 1.200×).

Thermogravimetric analysis showed organic matter volatilisation from 100 to 550°C (Figure 9), thus indicating organic compounds with distinct thermal stabilities. The mass stabilisation was attained at temperatures higher than 550°C, and this behaviour can be attributed to aluminosilicates. In turn, the electron micrograph (Figure 10) also illustrates the natural porosity of the sediment, pointing to favourable adsorptive conditions and corroborating the infrared spectrum and thermogravimetric profile.

Infrared, electron microscopy and thermogravimetric analyses were conducted on the sediment collected at point 5, because of their interesting structural characteristics, including high contents of clay and total organic matter (Table 7). However, these characteristics may vary over the different collections and sampling points.

Despite the desirable structural features of the sediments for metal adsorption, the exchangeable and pseudototal concentrations of Cd, Cu, Pb, and Zn were within the normal limits for most samples. This finding reinforces the absence of significant pollution sources concerned with the evaluated analytes.

## 5. Conclusions

The analyses of water and sediment samples from the Jacuípe River's estuary revealed good environmental conditions in relation to the different physical-chemical parameters, despite the large regional development in terms of population growth and industrial diversification.

 Concerning the water compartment, the nitrite and nitrate levels point to an absence of significant quantities of biodegradable organic material, and adequate oxygenation levels also support this conclusion. The normal levels of soluble phosphate indicate that there is no appreciable source of waste containing detergents. This last result, along with normal concentrations of nitrate, shows an aquatic ecosystem preserved from eutrophication. It must be noted that this environmental panorama was observed, despite increasing human pressure promoted by real-estate development and tourism. The pH parameters and electrical conductivity were also classified as normal when checked against Brazilian environmental legislation and other works.

 The reduced total concentrations of Cd(II), Cu(II), Pb(II), and Zn(II) in the water point to an absence of continuous sources of discharges, especially of industrial origin, regardless of the proximity to the Camaçari petrochemical complex.

 For the sediments, the mineralogical composition, the morphological aspect of particles, the infrared spectrum, as well as the total levels of organic matter helped in elucidating the sediment's potential adsorptive capacity. The decrease in clay levels after the fifth sampling site is consistent with the geographical characteristics of estuarine areas.

 All the samples displayed exchangeable levels of Cd, Cu, Pb, and Zn well below those established in the literature. The exception to this behaviour was found in some few results for cadmium. The presence of higher levels of all four metals in the pseudototal fraction is coherent with the theoretical expectations, although most of the pseudototal results are below the maximum allowable levels.

 Finally, this study updated and increased the database on the environmental quality of the Jacuípe River Estuary, an area of great ecological importance to the preservation of tropical ecosystems on the Brazilian northeastern coast, and of pronounced economic importance to the Bahia State.

## Figures and Tables

**Table 1:** Dates (month/year) of the water and sediment sampling campaigns.

Sampling number	Water	Sediment
1	03/2007	03/2007
2	05/2007	07/2007
3	06/2007	08/2007
4	07/2007	09/2007
5	08/2007	10/2007
6	09/2007	03/2008
7	10/2007	06/2008
8	11/2007	07/2008
9	03/2008	—
10	04/2008	—
11	06/2008	—
12	08/2008	—
13	09/2008	—

**Table 2:** Physical-chemical parameters of water collected in the Jacuípe River's estuary.

Date (sampling)	Point	Temperature (°C)	pH		Conductivity (mS cm^−1^)		DO (mg L^−1^)
		S	S	D	S	D	S
03/2007 (1)	1	29.3	7.79	7.89	40.60	40.00	5.5
	2	27.2	7.12	7.88	10.20	39.90	7.8
	3	28.6	6.73	6.82	1.46	1.57	8.6
	4	28.4	6.77	6.88	0.20	0.21	8.8
	5	28.1	6.79	6.94	0.12	0.12	9.6

05/2007 (2)	1	28.5	7.45	7.24	7.45	2.52	6.3
	2	27.0	7.32	7.20	3.24	1.90	7.5
	3	27.6	7.22	7.24	1.22	0.62	8.4
	4	26.8	7.14	7.20	0.21	0.21	8.6
	5	27.2	7.19	7.25	0.22	0.18	8.6

06/2007 (3)	1	27.2	7.30	7.10	1.33	0.74	3.9
	2	26.6	6.80	7.00	0.93	0.43	7.6
	3	30.1	6.80	7.00	0.36	0.28	4.9
	4	27.5	7.20	7.20	0.16	0.17	5.1
	5	28.4	7.00	7.00	0.16	0.16	8.0

07/2007 (4)	1	28.2	6.00	6.20	6.92	7.64	8.3
	2	26.7	6.30	6.40	4.43	5.21	8.8
	3	26.5	7.00	7.10	0.97	1.21	7.6
	4	26.6	7.80	7.70	0.17	0.14	6.0
	5	27.5	8.00	7.80	0.13	0.18	9.1

08/2007 (5)	1	27.5	5.60	6.20	43.00	43.00	8.9
	2	26.8	7.30	6.70	1.20	39.40	9.4
	3	26.4	7.00	6.70	19.56	19.74	8.5
	4	27.3	7.10	7.20	6.93	9.85	8.2
	5	27.0	7.10	6.90	7.95	15.49	6.9

09/2007 (6)	1	26.0	7.43	7.35	22.20	22.10	8.8
	2	25.6	7.19	7.20	17.11	17.21	7.5
	3	26.5	6.87	6.79	5.68	6.14	9.3
	4	27.8	6.60	6.56	0.45	0.47	9.6
	5	26.8	6.50	6.60	0.38	0.40	8.7

10/2007 (7)	1	28.2	6.00	6.70	29.00	30.90	7.2
	2	27.3	7.30	7.00	17.19	29.50	7.9
	3	28.2	6.90	6.90	16.79	23.50	7.4
	4	28.0	7.10	6.80	4.28	6.83	7.7
	5	27.5	7.10	6.90	3.64	4.97	8.1

11/2007 (8)	1	27.7	7.90	7.90	48.60	48.50	7.9
	2	28.1	8.00	8.10	46.80	47.40	6.5
	3	28.4	8.00	8.10	41.30	42.80	7.1
	4	27.9	8.00	8.00	0.20	0.21	7.0
	5	27.9	7.90	7.90	0.12	0.12	6.8

03/2008 (9)	1	28.4	7.37	7.36	48.60	48.50	7.9
	2	29.4	7.39	7.28	46.80	47.40	6.5
	3	29.6	7.21	7.36	41.30	42.80	7.1
	4	28.7	6.73	6.87	0.20	0.21	7.0
	5	29.3	6.67	6.89	0.12	0.12	6.8

04/2008 (10)	1	28.5	7.48	7.62	41.60	42.80	6.4
	2	29.0	7.66	7.69	16.10	38.90	8.8
	3	29.1	7.59	7.46	1.53	1.83	8.1
	4	29.0	7.80	7.57	0.28	0.32	6.1
	5	29.3	7.50	7.31	0.14	0.15	6.1

06/2008 (11)	1	27.8	7.87	7.68	35.20	35.00	8.4
	2	28.1	7.45	7.70	5.87	32.70	7.2
	3	28.7	7.30	7.60	21,70	32.20	6.9
	4	28.2	7.21	7.03	8.47	9.51	8.0
	5	28.5	6.90	6.98	6.63	8.87	7.0

08/2008 (12)	1	25.1	7.54	7.64	3.80	36.80	8.4
	2	24.6	7.51	7.47	9.16	35.40	8.2
	3	23.7	7.15	7.28	31.00	33,90	8.5
	4	22.7	6.76	7.42	25.00	37.30	8.7
	5	22.7	6.76	7.15	24.50	30.20	8.7

09/2008 (13)	1	26.4	7.90	7.97	27.40	27.40	8.0
	2	26.6	7.74	7.81	22.00	22.10	8.5
	3	26.7	7.40	7.40	9.85	9.95	8.5
	4	26.4	7.14	7.05	0.49	0.55	8.0
	5	26.3	7.06	7.03	0.38	0.41	7.9

S: surface and D: deep.

**Table 3:** Concentrations (mg L^−1^) of nitrite (N-NO_2_^−^), nitrate (N-NO_3_^−^), and soluble phosphate (P-PO_4_^3−^) in water samples collected from the Jacuípe River's estuary, *n* = 3*.

Date (sampling)	Point	Nitrite (N-NO_2_^−^)		Nitrate (N-NO_3_^−^)		Phosphate (P-PO_4_^3−^)**	
		S	D	S	D	S	D
03/2007 (1)	1	<0.004***	<0.004	0.03	0.09	<0.02*	<0.02*
	2	0.02	<0.004	0.07	0.07	<0.02	<0.02
	3	0.01	0.02	0.07	0.06	<0.02	<0.02
	4	0.02	0.03	0.07	0.04	<0.02	<0.02
	5	0.02	0.02	0.06	0.05	<0.02	<0.02

05/2007 (2)	1	0.01	0.01	0.12	0.12	0.09	0.12
	2	0.01	0.01	0.05	0.12	0.08	0.11
	3	0.01	0.02	0.11	0.13	0.11	0.09
	4	0.01	0.01	0.13	0.15	0.03	0.03
	5	0.02	0.01	0.10	0.14	0.08	0.03

06/2007 (3)	1	0.01	0.02	0.11	0.09	0.11	0.14
	2	0.01	0.02	0.09	0.09	0.09	0.14
	3	0.03	0.02	0.09	0.10	0.16	0.16
	4	0.02	0.01	0.10	0.09	0.14	0.14
	5	0.02	0.01	0.10	0.10	0.13	0.14

07/2007 (4)	1	0.01	0.01	0.07	0.09	0.06	0.07
	2	0.01	0.01	0.06	0.09	0.09	0.09
	3	0.01	0.01	0.06	0.09	0.09	0.09
	4	0.01	0.01	0.15	0.12	0.05	0.05
	5	0.01	0.01	0.13	0.15	0.04	0.05

08/2007 (5)	1	<0.004	<0.004	0.07	0.05	0.16	0.09
	2	<0.004	<0.004	0.06	0.05	0.06	0.08
	3	< 0.004	<0.004	0.06	0.05	0.10	0.12
	4	0.01	0.01	0.09	0.05	0.10	0.13
	5	0.01	0.01	0.08	0.07	0.15	0.10

09/2007 (6)	1	<0.004	<0.004	0.13	0.11	0.06	0.06
	2	<0.004	0.01	0.21	0.12	0.08	0.07
	3	0.01	0.01	0.14	0.14	0.08	0.08
	4	0.01	0.01	0.11	0.10	0.05	0.21
	5	0.01	0.01	0.12	0.10	0.04	0.04

10/2007 (7)	1	<0.004	<0.004	0.01	0.09	0.22	0.06
	2	<0.004	<0.004	0.06	0.09	0.04	0.08
	3	<0.004	0.01	0.07	0.08	0.06	0.09
	4	0.01	0.01	0.05	0.09	0.06	0.06
	5	0.01	0.01	0.07	0.10	0.06	0.06

11/2007 (8)	1	0.011	0.015	0.11	0.10	0.12	0.12
	2	0.015	0.011	0.10	0.11	0.12	0.14
	3	0.012	0.013	0.11	0.12	0.13	0.13
	4	0.015	0.015	0.11	0.10	0.10	0.15
	5	0.016	0.015	0.11	0.11	0.12	0.13

03/2008 (9)	1	<0.004***	<0.004	0.07	0.09	0.02	0.03
	2	0.005	0.01	0.08	0.09	0.03	0.03
	3	0.005	0.01	0.08	0.10	0.05	0.04
	4	0.01	0.01	0.10	0.12	0.03	0.03
	5	0.01	0.01	0.09	0.08	0.03	0.03

04/2008 (10)	1	<0.004	<0.004	0.07	0.08	0.03	0.02
	2	<0.004	<0.004	0.07	0.14	0.04	<0.02*
	3	0.01	0.01	0.30	0.32	0.09	0.05
	4	0.01	0.01	0.20	0.33	0.04	0.03
	5	0.01	0.01	0.20	0.24	0.04	0.03

06/2008 (11)	1	<0.04	<0.004	0.12	0.01	0.03	0.03
	2	<0.004	<0.004	0.09	0.09	<0.02	0.04
	3	0.008	0.010	0.09	0.10	0.07	0.05
	4	0.005	0.01	0.01	0.09	0.03	0.03
	5	0.010	0.02	0.09	0.09	0.03	0.03

08/2008 (12)	1	<0.004	<0.004	0.07	0.10	0.03	0.04
	2	<0.004	<0.004	0.09	0.14	<0.02	0.05
	3	0.01	0.013	0.07	0.09	0.07	0.06
	4	0.008	0.01	0.10	0.10	0.04	0.03
	5	0.010	0.01	0.08	0.08	0.02	0.08

09/2008 (13)	1	<0.004	<0.004	0.08	0.09	0.03	0.03
	2	<0.004	<0.004	0.01	0.10	0.03	<0.02
	3	0.01	0.01	0.13	0.10	0.03	<0.02
	4	0.01	0.01	0.11	0.09	0.03	0.03
	5	0.01	0.01	0.10	0.10	0.03	0.04

S: surface and D: deep. *In order to simply the data exposition, the relative standard deviations were omitted, but all of these values were smaller than 10%. **Soluble phosphate. ***Limit of detection as 3*σ* [24].

**Table 4:** Exchangeable concentrations of Cd, Cu, Pb, and Zn (mg kg^−1^) in sediments collected from the Jacuípe River's estuary, *n* = 5.

Date (sampling)	Point	Cd	Cu	Pb	Zn
03/2007 (1)	1	<0.05*	10,1 ± 1.0	<5.0*	<1.3*
	2	<0.05	16.2 ± 0.7	<5.0	4.8 ± 0.2
	3	<0.05	13.9 ± 0.8	<5.0	29.2 ± 1.0
	4	<0.05	8.6 ± 0.3	<5.0	43.7 ± 0.2
	5	<0.05	8.7 ± 0.7	<5.0	23.1 ± 1.2

07/2007 (2)	1	1.1 ± 0.04	2.4 ± 0.03	<5.0	<1.3
	2	1.1 ± 0.01	2.5 ± 0.02	<5.0	4.7 ± 0.2
	3	1.2 ± 0.01	15.9 ± 0.1	<5.0	26.4 ± 0.1
	4	1.1 ± 0.01	44.6 ± 0.4	<5.0	43.4 ± 0.1
	5	1.3 ± 0.03	10.8 ± 0.2	<5.0	17.8 ± 1.4

08/2007 (3)	1	1.2 ± 0.01	2.2 ± 0.03	<5.0	4.0 ± 0.1
	2	1.1 ± 0.01	2.1 ± 0.04	<5.0	2.0 ± 0.04
	3	1.2 ± 0.01	2.1 ± 0.02	<5.0	2.5 ± 0.05
	4	1.2 ± 0.02	4.2 ± 0.2	<5.0	6.0 ± 0.2
	5	1.1 ± 0.01	11.1 ± 0.4	<5.0	<1.3

09/2007 (4)	1	<0.05	2.3 ± 0.03	<5.0	3.1 ± 0.1
	2	<0.05	2.4 ± 0.1	<5.0	<1.3
	3	<0.05	5.4 ± 0.04	<5.0	19.9 ± 0.1
	4	<0.05	6.5 ± 0.2	<5.0	17.2 ± 0.2
	5	<0.05	5.8 ± 0.1	<5.0	12.3 ± 0.3

10/2007 (5)	1	<0.05	<1.3*	<5.0	<1.3
	2	<0.05	<1.3	33.4 ± 4.0	<1.3
	3	<0.05	4.6 ± 0.2	<5.0	21.0 ± 0.2
	4	<0.05	6.1 ± 0.2	22.8 ± 0.8	19.3 ± 1.0
	5	<0.05	3.4 ± 0.3	12.2 ± 1.5	<1.3

*Limit of detection as 3*σ* [24].

**Table 5:** Pseudototal concentrations of Cd, Cu, Pb, and Zn (mg kg^−1^) in some sediment samples collected from the Jacuípe River's estuary, *n* = 5.

Date	Point	Cd	Cu	Pb	Zn
09/2007	1	8.8 ± 0.7	42.5 ± 0.9	10.6 ± 0.3	7.5 ± 0.5
	2	14.9 ± 1.1	48,7 ± 1,3	22.3 ± 1.7	4.4 ± 0.1
	3	13.4 ± 0.9	33.3 ± 1.6	37.4 ± 1.1	30.3 ± 1.1
	4	15.7 ± 0.8	27.3 ± 0.5	30.2 ± 0.6	20.4 ± 0.6
	5	19.4 ± 0.3	22.4 ± 0.8	27.9 ± 1.4	18.8 ± 0.9

03/2008	1	12.7 ± 0.9	66.3 ± 2.1	36.2 ± 1.3	6.3 ± 0.2
	2	10.9 ± 0.8	71.9 ± 2.7	28.3 ± 0.8	8.7 ± 0.7
	3	11.1 ± 0.7	84.0 ± 3.3	59.9 ± 1.7	34.4 ± 1.2
	4	14.5 ± 0.9	39.5 ± 2.0	38.5 ± 1.2	10.4 ± 1.0
	5	12.6 ± 0.8	45.3 ± 2.4	48.3 ± 1.0	12.7 ± 0.6

06/2008	1	5.7 ± 0.1	86.1 ± 0.9	19.4 ± 1.6	8.8 ± 0.1
	2	11.4 ± 0.7	78.1 ± 1.7	28.1 ± 1.1	6.1 ± 0.4
	3	13.3 ± 0.9	77.2 ± 0.8	68.2 ± 2.4	7.9 ± 0.7
	4	14.7 ± 0.2	59.3 ± 1.2	82.4 ± 0.9	35.7 ± 1.7
	5	15.2 ± 1.0	42.9 ± 0.7	43.0 ± 1.5	22.3 ± 1.0

07/2008	1	8.3 ± 0.4	47.0 ± 1.9	14.3 ± 0.4	15.2 ± 0.6
	2	11.3 ± 0.9	64.6 ± 3.6	27.7 ± 1.8	11.3 ± 0.9
	3	16.7 ± 0.2	41.1 ± 5.2	30.4 ± 1.8	16.6 ± 0.4
	4	12.8 ± 1.0	50.9 ± 3.2	44.8 ± 1.5	12.6 ± 0.5
	5	13.9 ± 1.0	54.5 ± 2.2	52.3 ± 1.1	22.8 ± 1.3

**Table 6:** Positions 2*θ* for minerals in the silt fraction of the sediment collected at sampling point 3, in March of 2007 [25–27].

Mineral (chemical formula)	Positions 2*θ*
Kaolinite (Al_2_Si_2_O_5_(OH)_4_)	14.2139 and 28.8365
Feldspar (CaAlSi_3_O_8_, KAlSi_3_O_8_ or NaAlSi_3_O_8_,)	31.93
Illite [general formula: K_x_(Al_2_)(Si_4-x_ Al_x_)O_10_ (OH)_2_]	10.1354 and 20.5040
Quartz (SiO_2_)	24.1458 and 30.9085

**Table 7:** Clay and total organic matter levels (%, m/m) of sediments collected in March of 2007.

Sampling point	Clay content	Total organic matter content (*n* = 3)
1	ND*	1.7 ± 0.4
2	ND	2.3 ± 0.1
3	8.8	6.7 ± 0.2
4	10.7	6.6 ± 0.1
5	12.0	7.8 ± 0.4

*Not detected.
